# The Diagnostic Accuracy of the Paris System for Reporting Upper Urinary Tract Cytology: The Atypical Urothelial Cell Conundrum

**DOI:** 10.3390/cancers17071097

**Published:** 2025-03-25

**Authors:** Mohamed A. Mansour, Luka Ozretić, Soha El Sheikh

**Affiliations:** 1 Department of Surgery, East & North Hertfordshire NHS Trust, Stevenage SG1 4AB, UK; mohamed.mansour7@nhs.net; 2 Department of Cellular Pathology, Royal Free London NHS Foundation Trust, London NW3 2QG, UK; luka.ozretic@nhs.net; 3 Research Department of Pathology, University College London (UCL) Cancer Institute, London WC1E 6DD, UK

**Keywords:** upper urinary tract, urothelial carcinoma, The Paris System for urine cytology, urine cytology

## Abstract

Background: The diagnosis of upper urinary tract urothelial carcinoma (UTUC) in cytological specimens is challenging, particularly the designation of atypical urothelial cells (AUC). The application of the Paris System for Reporting Urinary Cytology (TPS) has improved the performance of lower tract urothelial carcinoma specimens but has shown variable results in upper tract specimens, which are frequently instrumented. Methods: This retrospective study analysed upper tract selective cytology samples from January to December 2023. Samples were classified under TPS 2.0 categories. Histological specimens were used where available as the gold standard to calculate statistical metrics including sensitivity, specificity, positive predictive value (PPV), and negative predictive value (NPV). Results: Out of 122 samples, 12.2% were considered non-diagnostic or insufficient, with 13.1% designated as Non-high-grade urothelial carcinoma (N-HGUC), 53.3% as atypical urothelial cells (AUC) and 21.3% as positive/suspicious for HGUC. Histopathological correlation was available for 48.7% of cases. The risk of malignancy was: NHGUC (0%), AUC (47%) and HGUC (77.7%). The highest PPV was for HGUC (78%), with a diagnostic accuracy of 81.3% and specificity of 88%. In contrast, AUC had a PPV of 47% in instrumented and non-instrumented samples, rising to 58% in combination with the HGUC category. Conclusion: TPS 2.0 is an effective tool with excellent diagnostic accuracy for HGUC and in excluding malignancies in the N-HGUC category, but in our hands, the high rates of the AUC category, together with the low PPV, remain a major challenge and an obstacle to the correct stratification of patients with UTUC.

## Simple Summary

Tumours that arise in the urinary system above the level of the urinary bladder are difficult to diagnose through radiology alone. Endoscopic examination, together with taking a cellular sample or tissue sample, is required to confirm whether there is a tumour. This study aims to determine both the diagnostic accuracy of cellular samples compared to tissue biopsies and how frequently they can correctly predict the presence or absence of cancer using a recently proposed classification system. We found that the classification system works well in terms of excluding the presence of tumours and in confirming the presence of aggressive tumours, but that it is less reliable in differentiating reactive changes due to inflammation from less aggressive tumours. Our study thus adds to knowledge about how best to use cellular samples and their classification system.

## 1. Introduction

Upper urinary tract urothelial carcinoma (UTUC), which arises in the ureter (including its orifice), renal pelvis or renal calyces, is uncommon and represents 5–10% of all urothelial carcinomas (UC) [1]. In addition to known risk factors of UC such as smoking and intake of aristolochic acid, some hereditary conditions such as Lynch syndrome are also risk factors for the disease [2,3]. Because of the difficulty in its diagnosis, UTUC tends to present in later stages and has a worse prognosis compared to urothelial carcinomas arising in the bladder [4].

The recommended imaging modality for the diagnostic workup of UTUC is multiphase computed tomography urography (CTU), but this technique is less sensitive for small or flat urothelial lesions that do not cause significant thickening of the urothelial lining [5]. Current guidelines from the European Association of Urology (EAU) [6] and the American Urological Association (AUA) [7] strongly recommend diagnostic ureteroscopy (URS) and upper tract cytology as integral parts of the standard workup for patients with suspected UTUC, and advise that this should ideally be performed selectively and before injection of contrast medium.

Flexible URS allows exploration and inspection of the upper urothelial tract and the performance of biopsies that can be targeted to any suspected lesions. However, URS is a traumatic invasive and expensive procedure that cannot be applied on a frequent basis. Cytology, on the other hand, is simple, cost-effective, and widely used method of assessment of exfoliated urothelial cells. Cytological examination of voided urine can be used to assess the entire urothelial tract, but it is more reliable for the diagnosis of lower urinary tract UC compared to UTUC [8].

Selective cytology from each ureter (whether selective urine retrieval or barbotage cytology, termed ureteric washings) and biopsy are critical in the stratification of patients with UTUC into low- or high-risk disease, where patients with low-risk disease are offered kidney-sparing endoscopic resection and/or thermal ablation by URS, while patients with high-risk disease are indicated for radical nephroureterectomy (NU) [9].

The Paris System for Reporting Urinary Cytology (TPS) [10] provides a standardised cytomorphologic reporting system for urinary cytology specimens. In its 2022 update (TPS 2.0), it included a section on UTUC and focused its diagnostic approach on the identification of high-grade urothelial carcinoma (HGUC), with less emphasis on the detection of low-grade (LG) urothelial neoplasms, which are classified either as atypical urothelial cells (AUC) or non-HGUC [11,12]. The diagnosis of LGUC in urine cytology specimens is generally difficult, and the performance of urine cytology in terms of detecting all urothelial carcinomas in the lower tract is typically worse than that for detecting only HG lesions [11].

In TPS 2.0, the cytomorphological criteria for UTUC are similar to those described for lower tract UC, including a nucleocytoplasmic (N/C) ratio at or above 0.7, marked nuclear membrane irregularities, coarse chromatin and moderate to severe hyperchromasia seen in at least 10 well-preserved malignant cells.

In this study, we investigate the diagnostic utility of upper tract cytology samples in the detection of UTUC, according to the second edition of TPS. We assess diagnostic accuracy, specificity, sensitivity, PPV and NPV using upper tract biopsies and NU as the gold standard.

## 2. Materials and Methods

This retrospective cytologic-histologic correlation study was conducted in compliance with the STARD criteria [13]. First, we searched for urinary cytology specimens that included the terms “upper tract”, “ureter” and “ureteric/pelvic washing”, dated from January to December 2023, in our LIMS pathology database. We also retrieved patient demographics, clinical details and cytological and histopathological reports. Follow up data were retrieved, and any subsequent cytology or histology sample was recorded.

The search terms we used retrieved some voided urine specimens from patients who were under surveillance after NU or had prior endoscopic ablation of UTUC, since these patients’ reports contained the word “ureter”. These cases were excluded, and the remaining specimens were further analysed.

To facilitate the statistical analysis, we divided the cytology samples into four categories: (1) non-diagnostic, (2) negative for HGUC (N-HGUC), (3) atypical urothelial cells (AUC), and Suspicious or Positive for HGUC (4) (HGUC). In parallel, we analysed cytology categorising AUC also as positive.

The concomitant or subsequent corresponding biopsies/NU taken within 12 months of the cytology were used to determine diagnostic accuracy and for statistical analysis. The biopsies were categorised into benign, LGUC and HGUC; the latter two include carcinoma in situ (CIS) and high grade papillary/solid invasive UC.

Statistical analysis:
Utilising the histological diagnosis in biopsies or NU as the gold standard, the sensitivity, specificity, PPV, NPV and overall accuracy were calculated as follows:
Sensitivity: true positive/(true positive + false negative).
Specificity: true negative/(true negative + false positive).
PPV: true positive/(true positive + false positive).
NPV: true negative/(true negative + false negative).

In addition, we calculated the risk of malignancy for each category by dividing the number of histologically confirmed malignant cases by the total number of cases in that diagnostic category and the diagnostic accuracy: (true positive + true negative)/total cases analysed. Chi Square test with Yates correction was used to compare the frequency of AUC cytology in instrumented and non-instrumented groups. All statistical analyses were performed using IBM SPSS Statistics for Windows, version 26.0 (IBM Corporation, New York, NY, USA). To visualise and interpret the results of probability calculations, we used the Test Accuracy tool developed by the NIHR Diagnostic Evidence Co-operative, Newcastle [14].

## 3. Results

### 3.1. Clinical and Cytological Findings

The search retrieved a total of 188 cytology specimens, of which 66 voided urine specimens were excluded, leaving 122 cytology specimens (Supplementary Table S1). Of these 122 samples, 91 were from the ureter and 31 from kidney/renal pelvis. The specimens belonged to 104 patients, 55 of which were males and 49 were females, with a median age of 73 years (range 36 to 92 years).

**Figure 1:**
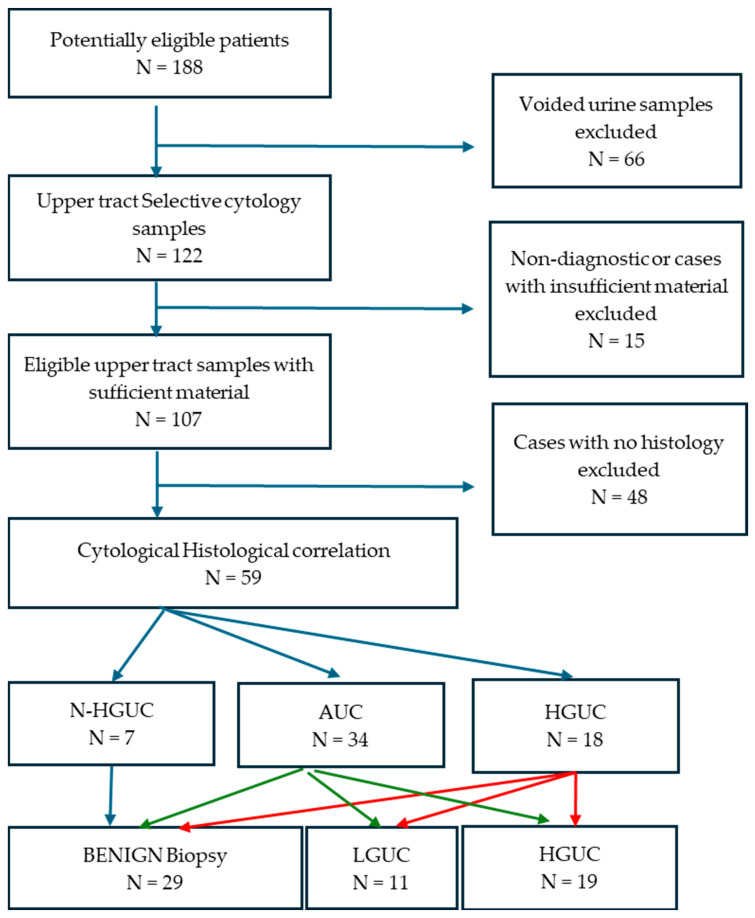
Data flow diagram highlighting the study design, and how cases were collected, criteria for exclusion, final number of cases analysed and main findings. Please also see Table 2. Abbreviations: N-HGUC—negative for HGUC. AUC—atypical urothelial cells; HGUC—high-grade urothelial carcinoma; LGUC—Low grade urothelial carcinoma.

TPS 2.0 comprises six diagnostic categories (non-diagnostic, N-HGUC, AUC, suspicious for HGUC, HGUC and other malignancies), but in this study, we classified our 122 cases into four categories by combining the suspicious for HGUC (only three cases) and HGUC cases into a single diagnostic category in our cohort (Figure 1).

Ureteric washings were the most frequently encountered sample (45%) and included one from a transplanted ureter. Renal pelvis/kidney washing was the least frequent sample (7.3%) (Table 1). Most of the cases were designated as AUC (53.3%) or HGUC (21.3%), and only 13.1% of cases were labelled as N-HGUC. The proportion of HGUC cases was highest in ureteric urine and renal pelvis urine (33.3% for both) and lowest in ureteric washings (12.7%).

**Table 1:** Distribution of types of upper tract cytological specimens and the diagnostic categories.

	Non-Diagnostic	N-HGUC	AUC	HGUC	Total
Ureteric Wash	10	10	28	7	55 (45%)
Ureteric urine	3	3	18	12	36 (29.5%)
Renal pelvis wash	2	1	3	3	9 (7.3%)
Renal pelvis urine	0	2	16	4	22 (18%)
Total	15 (12.3%)	16 (13.1%)	65 (53.3%)	26 (21.3%)	122

Abbreviations: AUC—atypical urothelial cells; HGUC—high-grade urothelial carcinoma; N-HGUC—negative for HGUC.

Next, we excluded 15 cytology cases that fell into the non-diagnostic category (12.3%): 10 ureteric washings, 3 ureteric urine and 2 renal pelvis urine), and another 48 cases that did not have corresponding biopsies/NU from the cytologic-histologic correlation, as they could not be classified as either true positive or negative.

### 3.2. Cytological-Histological Correlation

Histopathological tissue samples were available for 59 cases (48.7%). Twenty five of 59 (42.3%) cases were ureteric washings, with ureteric urine and kidney urine comprising 14 (23.7%) and 16 (27.1%) samples, respectively. Kidney washings comprised the lowest proportion of cytological samples at 4 (6.7%).

For the biopsies, 48 were ureteric or renal pelvis biopsies (81.4%), and the remaining 11 cases (18.6%) were NUs. Nine of the 11 NUs were for HGUC cytology with concordant diagnostic imaging, and 2 cases had AUC cytology in a non-functioning kidney, so a decision was made to perform a diagnostic NU.

In the majority of cases, the tissue sample used in this correlation was a biopsy taken at the same time as the URS (median = 0 days, range = 0–175 days). Some patients proceeded directly to NU (median = 34 days, range 23–131 days). For the entire cohort, the mean was 27 days (+/−40 days).

Histologically, 29 out of 59 cases were benign, 11 were LGUC and 19 were HGUC. Reassuringly, all of the cases designated N-HGUC were benign on biopsy with no false negative cases. The risk of any urothelial malignancy, whether LGUC or HGUC, after an AUC diagnosis was 47%, with a diagnostic accuracy of 57.3%. In contrast, the risk after a cytological HGUC diagnosis was 77.7%, with a diagnostic accuracy of 81.3%, but this was specifically for a histologically HGUC diagnosis. The cytological and histopathological diagnoses of the 59 cytology and histology samples in the different categories are summarised in Table 2.

**Figure 2:**
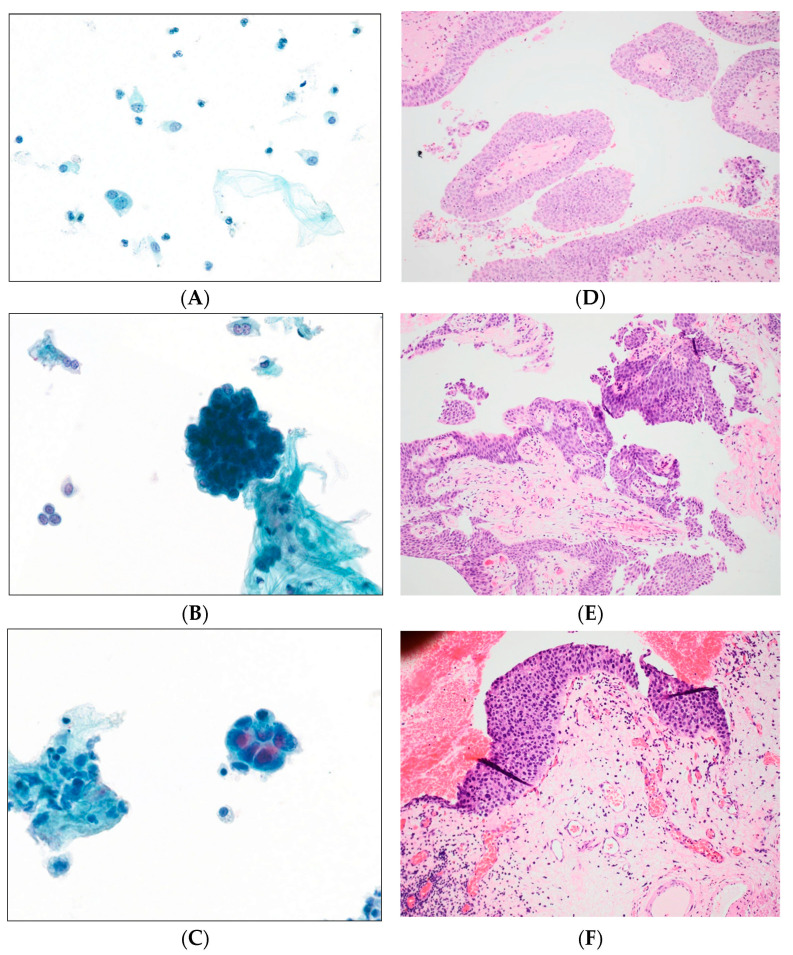
Examples of upper tract cytology samples according to the TPS 2.0, and the corresponding histological findings on biopsy. (**A**) No High-grade urothelial carcinoma (N-HGUC): Benign urothelial cells. Singly scattered cells with low N:C ratio, round nuclei and no profound chromatin abnormalities. Note the inflammatory cells in the background (Ureteric washing, ThinPrep, high magnification—40×). (**B**) Atypical urothelial cells (AUC). The cells demonstrate increased N:C ratio (<0.7), enlarged nuclei and mild nuclear contour irregularities (Ureteric washing, ThinPrep, high magnification—40×). (**C**) High-grade urothelial carcinoma (HGUC). A group of abnormal cells with enlarged irregular nuclei, N:C ratio of 0.7, nuclear hyperchromasia and coarse chromatin (Ureteric urine, ThinPrep, high magnification—40×). (**D**) Benign urothelium of the renal pelvis may be thrown into papillaroid folds and show reactive changes if inflamed, (Magnification—20×). Detached degenerate cells may be confused for AUC on cytology (arrow). (**E**) Low grade papillary urothelial carcinoma with nuclear atypia and loss of polarity. The cores are fibrous. (Magnification—40×). (**F**) High grade urothelial carcinoma in the form of carcinoma in situ (CIS) with severely atypical nuclei and increased mitotic activity. (Magnification—40×).

Examples of the various TPS 2.0 diagnostic categories and their corresponding biopsies are shown in Figure 2.

We then analysed the sensitivity, specificity, PPV and NPV of AUC and HGUC to predict either benign, LGUC or HGUC status upon biopsy. The results are shown in Table 3.

**Figure 3:**
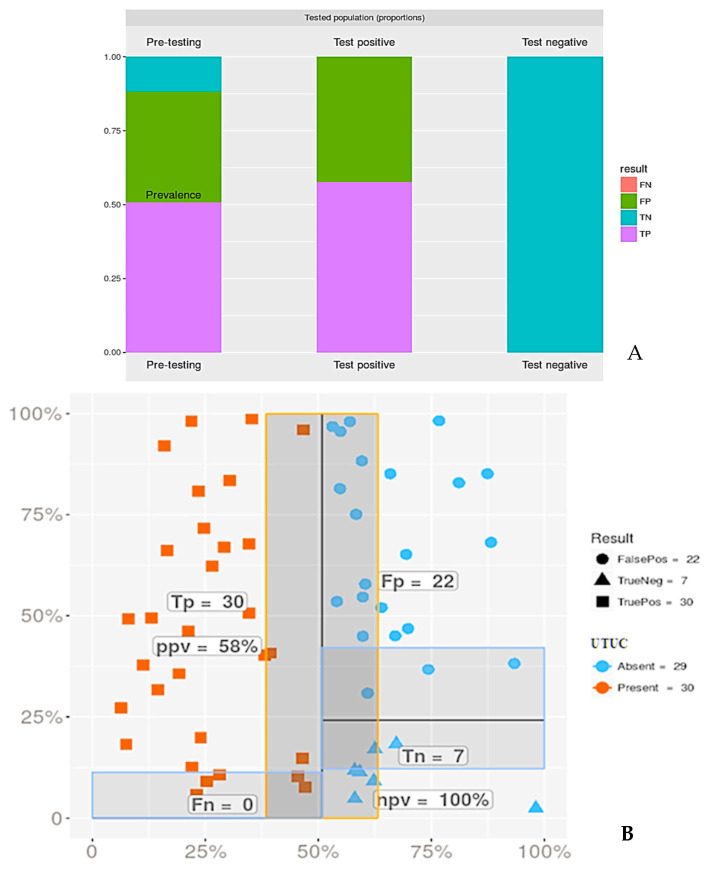
The overall diagnostic accuracy of TPS 2.0 for predicting UTUC in our cohort. (**A**). Bar Chart and (**B**), Scatter plot to visualise the population of patients tested for UTUC, coded to show the proportions of the population who tested positive and who had UTUC (TP), tested negative and did not have UTUC (TN), or tested positive and did not have UTUC (FP). There were no cases that tested positive and subsequently had UTUC (FN). Abbreviations: FN—false negative; NPV—negative predictive value; PPV—positive predictive value; TN—true negative; TP—true positive.

Overall, the PPV of TPS 2.0 in diagnosing UTUC of any grade was 58%, when AUC and HGUC were considered together (Figure 3), with a higher PPV if only the HGUC category was considered. The rate of any malignancy after an AUC diagnosis was 55%, and this PPV was reduced for LGUC on biopsy to 29%, despite the fact that most of the LGUC biopsies were reported as AUC. There were no false negative cases.

### 3.3. Instrumentation and AUCs

AUC, as a category, showed very low PPV for LGUC (29%) with a high NPV of 96%. When combined with the HGUC category, it reduced the PPV of HGUC from 78% to 58%. To investigate whether there was an increase in the frequency of the AUC category in instrumented samples obtained by barbotage compared to non-instrumented samples (catch of selective ureteric urine), we analysed these groups separately. There were 17 AUC cases in 29 washings (instrumented samples) and 17 AUC cases in 30 ureteric/renal pelvis urine samples (non-instrumented) (Supplementary Table S1). No statistically significant difference in the frequency of AUC cytology in the two groups was detected (χ^2^ = 0.0125; *p* > 0.05). The sensitivity, specificity, PPV and NPV of AUC in instrumented versus non-instrumented samples are shown in Table 3. Overall, greater NPV, sensitivity and specificity were obtained in ureteric washings compared to ureteric urine, but the PPV was similar (47%).

### 3.4. Follow Up

Follow up data were available for 50 out of 59 patients included in the histo-cytologic correlation arm of this study. The range of follow up was 11 to 22 months (median 13 months). All patients remained in the respective categories as shown in Table 2. No patients who were designated N-HGUC or AUC switched to the HGUC category, except for one patient who had an initial AUC with negative biopsy in 2023, then had a subsequent biopsy in 2024 which showed LGUC. The significance of this finding is uncertain, considering the long gap between the diagnostic URS cytology and positive biopsy (16 months).

Other than the 9 patients who proceeded directly to NU as their initial tissue diagnosis, there were 10 patients who were diagnosed as having HGUC upon biopsy. Five of these patients subsequently underwent a NU. Of the remaining 5 who did not undergo a NU, 3 were deemed unfit for surgery and had local disease control combined with external beam radiation therapy or immunotherapy. One patient was lost to follow up (concomitant malignancy) and another was deceased (Supplementary Table S1). For 11 patients with LGUC on biopsy, 4 underwent NU due to a large volume or multifocal disease, with conservative endoscopic management achieving local control in the remaining 7 patients.

## 4. Discussion

TPS 2.0 is a relatively novel classification system that establishes standardised diagnostic criteria to provide uniform reporting of urine cytology and improve risk stratification. A new chapter on urine cytology of the upper tract has been introduced in recognition of the specific challenges in establishing the diagnosis of UTUC.

In this study, we analysed all selective upper tract cytology specimens and the corresponding histology from a one-year period (2023) in a tertiary renal cancer centre. Our results show N-HGUC category has excellent NPV in terms of excluding UTUC, with none of the cases showing malignancy. The PPV of HGUC cytology category for the detection of HGUC in biopsy/NU was 72%, with a specificity of 88% and a sensitivity of 68%. The PPV of HGUC increased to 77% if the histological diagnosis was either LG or HGUC on biopsy/NU.

Pre-TPS, a meta-analysis that systematically reviewed literature from 2005 to March 2016 found that the overall sensitivity and specificity of upper urinary tract cytology for diagnosing UTUC was 53% (95% CI, 42.3–63.6) and 90% (95% CI, 85.4–93.2), respectively [15]. Some studies have reported that TPS had a stronger correlation with histological diagnosis in the lower tract compared with UTUC [16]. However, studies are emerging that show that the application of TPS for the diagnosis of HGUC in upper tract cytology has yielded sensitivity ranging from 19% to 82%, but with relatively high specificities, ranging from 86% to 100% and PPV ranging from 92% to 100% [17,18]

Overall, AUC cases account for 4–6% of voided urine specimens, with a risk of malignancy of 32.4–66.7% [19,20], but the rates of AUC cases reported in upper tract cytology have generally been higher (27–38%) [21,22,23].

In our study, our AUC rate was 53%, which is relatively high. One limitation of our study was that it did not include a comparison of AUC rates pre and post TPS 2.0 or an analysis of interobserver variability between cytologists and personal pathologist performance against the tissue diagnosis. Several studies have shown that the application of TPS 2.0 can reduce the AUC rate compared to pre-TPS 2.0 with post-TPS 2.0AUC rates ranging from 3.5% to 44.2% (median = 16%) [18]. In one study [24], the rate of AUC increased from 38.5% to 44.2% in the same institution after the application of TPS 2.0. The wide range of AUC categories in UTUC, together with the mixture of increased and decreased rates post TPS 2.0, suggest a possible degree of subjectivity in the interpretation of the cytologic criteria of AUC in different institutions. In TPS 1.0, interobserver agreement was lowest in the AUC category (κ = 0.178) [25], but there are no available studies for interobserver agreements using the relatively recent TP 2.0. To obtain meaningful data about interobserver variability in cytology findings and TPS 2.0 interpretation, a larger cohort of cases than those included in this study is required.

The PPV of AUC for the detection of any urothelial malignancy (LG or HG) in histological specimens was 55%, with a specificity of 50% indicating the relatively non-specific nature of this category. When analysed in relation to LGUC in biopsies/NU, the PPV was worse (at 29%) than when combined with HGUC. These figures indicate a possible overdiagnosis of the AUC category in our centre and the need for improvements, re-education and possible double reporting.

It is important for cytopathologists to recognise that AUC should not be used as a waste basket diagnosis. TPS 2.0 concentrates on detecting HGUC, which is linked to more aggressive disease that may be associated with local invasion and metastasis. It does not prioritise the detection of superficial non-invasive LGUC that are observable by urologists [10].

The diagnosis of UTUC remains a major challenge for cytopathologists due to its rarity and the frequent reactive cytomorphological changes introduced by the often-repeated instrumentation. The difficulty is compounded by the reported variable appearance of HGUC in the upper tract, where the cells may show smaller nuclei and less cytoplasm compared to lower tract UC [26]. They also often lack marked nuclear membrane irregularity [21] and may show hypochromatic rather than hyperchromatic nuclei [13,23].

One case in our cohort had AUC on cytology and an initial benign biopsy but showed LGUC in a subsequent URS tissue sample, highlighting the importance of adhering to the EAU and AUA guidelines [6,7] of clinical follow-up, with repeated cytology and biopsy, in patients suspected of having UTUC. This case also indicates that the use of URS biopsies as the gold standard for determining cytology performance in some of the cases may have been a limitation of our study; caution should therefore be exercised in the interpretation of the results in this subset. In contrast, NU is the ideal gold standard for this type of study, because the entire upper urothelial tract is amenable to histological examination and the presence of UTUC can then be confirmed or excluded with certainty.

Evaluation of cytological and histological material and obtaining the correct pathological diagnosis are dependent on the performance of an adequate URS. The overall diagnostic accuracy of URS is reported to range from 74–78%, but URS grade predictions based on the visual appearance of papillary UTUC with digital ureteroscopy are often incorrect in comparison with biopsy results [27]. There are also reports of URS biopsies missing 25% of lesions and that 65% of the cases were upstaged at the final histopathology [28].

So far, only routine cytological analysis is utilised to diagnose upper tract cytology specimens. Recent advances in our understating of the molecular underpinnings of urothelial carcinoma and rapid developments in the field of image analysis and artificial intelligence may offer ancillary tests or algorithms with the potential to enhance the accuracy of upper tract cytology and improve performance.

## 5. Conclusions

Due to its rarity, studies that specifically address the use of TPS 2.0 in the diagnosis of UTUC are infrequent. Our study adds to our knowledge about the performance of TPS 2.0 and supports its application in the diagnosis of UTUC. Our data highlight the strengths of TPS 2.0, both in terms of sensitivity and specificity, in diagnosing HGUC and in excluding the presence of HGUC if the case is reported as N-HGUC. It also points to its weakness in tackling the more frequent characterisation of cytological atypia in the upper tract, resulting in a high AUC category with low sensitivity and PPV. The limitations of TPS 2.0 in diagnosing LGUC are well documented, but more stringent application of the cytomorphologic criteria of TPS 2.0 and further refinement of its guidelines may help reduce the AUC rate to bring it to similar levels to those observed in the lower tract. In the future, the routine application of validated ancillary molecular tests may enhance the diagnostic accuracy of upper tract urothelial cytology.

## Figures and Tables

**Table 2:** The correlation between TPS 2.0 cytological criteria (rows) and the corresponding histological diagnosis, including the risk of malignancy and diagnostic accuracy of each category.

Cytological Category	Histological Results from Biopsy and NU				Risk of Any Malignancy *	ROHM	Diagnostic Accuracy **
	Benign	LGUC	HGUC	Total			
N-HGUC	7	0	0	7	0%	0%	62.7%
AUC	18	10	6	34	47%	17.6%	57.6%
HGUC	4	1	13	18	77.7%	72.2%	81.3%
Total	29	11	19	59			

* Risk of any malignancy includes both LG and HG malignancy. ** Diagnostic accuracy analyses comparable categories. i.e., N-HGUC for benign biopsy, AUC for LGUC and HGUC in cytology versus HGUC on biopsy. Abbreviations: AUC—atypical urothelial cells; HGUC—high-grade urothelial carcinoma; HG—High grade; LG—Low grade.

**Table 3:** Breakdown of diagnostic accuracy statistics of UT cytology using TPS 2.0 in 59 cases determined against the corresponding histological diagnosis.

Overall diagnostic accuracy of TPS 2.0 (AUC + HGUC cytology and malignant diagnosis *		
Statistic	Value	95% CI
PPV	0.58	0.53–0.63
NPV	1.00	0.59–1.00
Sensitivity	1.00	0.88–1.00
Specificity	0.24	0.1–0.44
HGUC cytology and HGUC on biopsy		
Statistic	Value	95% CI
PPV	0.72	0.52–0.86
NPV	0.85	0.75–0.92
Sensitivity	0.68	0.43–0.87
Specificity	0.88	0.73–0.96
HGUC cytology and malignant biopsy		
Statistic	Value	95% CI
PPV	0.78	0.57–0.90
NPV	0.61	0.52–0.69
Sensitivity	0.47	0.28–0.66
Specificity	0.86	0.68–0.96
AUC cytology and malignant biopsy		
Statistic	Value	95% CI
PPV	0.55	0.43–0.66
NPV	0.50	0.40–0.63
Sensitivity	0.55	0.36–0.73
Specificity	0.50	0.31–0.69
AUC cytology and LGUC biopsy		
Statistic	Value	95% CI
PPV	0.29	0.23–0.37
NPV	0.96	0.78–0.99
Sensitivity	0.91	0.59–1.00
Specificity	0.50	0.35–0.65
AUC + HGUC cytology in instrumented samples and malignancy biopsy		
Statistic	Value	95% CI
PPV	0.47	0.33–0.62
NPV	0.67	0.44–0.84
Sensitivity	0.67	0.35–0.91
Specificity	0.47	0.23–0.72
AUC + HGUC cytology in non-instrumented samples and malignancy biopsy		
Statistic	Value	95% CI
PPV	0.47	0.33–0.62
NPV	0.23	0.09–0.47
Sensitivity	0.44	0.22–0.69
Specificity	0.25	0.05–0.57

* Malignant biopsy includes both HGUC and LGUC. Abbreviations: AUC—atypical urothelial cells; HGUC—high-grade urothelial carcinoma; HG—High grade; LGUC—Low grade urothelial carcinoma.

## Data Availability

The original contributions presented in this study are included in the article and Supplementary Material. Further inquiries can be directed to the corresponding author.

## Supplementary Materials

The following supporting information can be downloaded at: https://www.mdpi.com/article/10.3390/cancers17071097/s1. The data used in this study are present in Supplementary Table S1: Clinicopathological data and URS cytology and biopsy results of the cohort used in this study.

## Abbreviations

The following abbreviations are used in this manuscript:

AUC	Atypical urothelial cells
LGUC	Low grade urothelial carcinoma
HGUC	High grade urothelial carcinoma
N-HGUC	Non high grade urothelial carcinoma
PPV	Positive predictive value
NPV	Negative predictive value
UTUC	Upper tract urothelial carcinoma
TPS 2.0	The Paris System for Reporting Urinary Cytology
CI	Confidence interval
EAU	European Association of Urology
AUA	American Urological Association
NU	Nephroureterectomy
URS	Ureteroscopy
STARD	Standards for Reporting Diagnostic accuracy studies
